# Observations upon Surgical Measures in Philadelphia

**Published:** 1892-08

**Authors:** 


### Editorial.

### OBSERVATIONS UPON SURGICAL MEASURES IN PHILADELPHIA.

A few comments upon surgical practice are suggested by a stay of two weeks in Philadelphia, and daily observations of operations by various prominent surgeons. The first notable feature is the general use of ether anæsthesia, with the exception of the employment of bromide of ethyl in certain cases by Dr. E. E. Montgomery. He follows in this, the lead of Chisholm, of Baltimore, who in a conversation at Detroit, urged upon me the safety and efficiency of this agent, not only in his specialty, but in general surgery. The results of Sims and Levis, some years ago, caused such fears of the consequences of bromide of ethyl inhalations, that few have since resorted to it. But I am assured by the above named operators, that this apprehension is without just foundation.

Again, it was observed that surgeons generally, and especially gynecologists in Philadelphia, employ simply sterilized water for all operations in which there is not already an infected or septic condition of the tissues involved, and no antiseptic is used in washing normal structures during an operation, in whatever part it may be. It was noted, however, that Ashhurst used a douche of a weak solution of bichloride of mercury, after excision of the knee joint, involved in suppurations with an extensive sulcus extending up between the muscles of the thigh. If bichloride washes are limited to septic surroudings, less trouble may be expected from poisoning, than occurs under the indiscriminate resort to this antiseptic agent.

In gynecological work, it was observed that the Prices use white silk ligatures in securing pedicles and pus tubes within the abdomen, while Montgomery employed cromatized cat-gut in these cases, as well as to close the external wound. Each of them make a point of suturing the intermuscular fascia separate from the deep through-and-through sutures which include the peritoneum. My individual experience favors the closure of the peritoneum with continuous cat-gut suture apart from the other tissues, so as to allow the natural mobility of this membrane in its union by first intention at the outset

It was observed in the Pennsylvania Hospital, that water dressings were still employed in the treatment of contused wounds and inflammatory conditions of the extremities, whereas the dry applications are generally adopted with good results. Since the fatal pneumonia in Stonewall Jackson's case, from the moisture caused by water dressing of his amputated arm, it has impressed most surgeons that this mode of treatment is more honored in the breach than by the observance.

The old time fracture box, and the resort to extension, by the weight and pulley, in fracture of the lower extremity, continues to be used in this institution. But I had the satisfaction to witness a very neat and efficient application of plaster of-Paris dressing with zinc plate stays, by Dr. Packard, in a fracture of the tibia. It was not made so thick and strong as I saw in New York some years ago applied by Dr. James R. Wood, who stood upon the plaster mould with his entire weight, to illustrate its strength, when I was taking the round of the hospital with him. The lighter plaster dressing can be made the better, and in this respect, the mode adopted by Dr. Packard fulfilled the indications admirably. The plan of suspension for fractures of the leg is also adopted with good results.

An impression is growing with the profession generally, that there is too much operating upon the abdominal cavity by gynecologists, and that the risks of oophorectomy and removal of tubes should be avoided by a resort to other modes of treatment, whenever practicable.

In the brief period of two weeks observation, it so turned out that I witnessed the fatal result of three abdominal sections, on account of purulent conditions involving the ovaries, tubes and peritoneum. These operations were in different places and by different parties, with such complications that they would almost certainly have terminated fatally without interference, but they seem to accentuate the caution against operating when it can be avoided.

Time and space will not allow further remarks at present.

J. McF. G.

